# Book Reviews

**Published:** 1821-06

**Authors:** 


### CRITICAL ANALYSES

### OF

### RECENT PUBLICATIONS, IN THE DIFFERENT BRANCHES OF MEDICINE AND SURGERY.

"I would have men know, that, though I reprehend the easie passing over of the causes of things

"by ascribing them to secret and hidden vertues and properties; (for this hath arrested and laid

"asleepe all true enquiry and indications;) yet I doe not understand but that, in the practical

"part of knowledge, much will be left to experience and probation, whereunto indication cannot

"so fully reach: and this not only in *specie*, but in *individuo*. Yet it was well said, *Vere scire*

"*esse per causas scire*."—BACON.

*Transactions of the Association of Fellows and Licentiates of the King's and Queen's College of Physicians in Ireland.* Volume III. 8vo. pp. 505. Cumming, Dublin; and Longman and Co. London. 1820.

THIS volume comprises several papers of considerable value; but, on the whole, its contents are much less interesting to the generality of medical men than those of either of the two former collections of the Transactions of the Association. A considerable proportion—above three hundred pages—of the present one is occupied by observations on the late Epidemy of Ireland, and Reports of the Fever-Hospitals of Dublin and Cork. So many Reports of this kind have been published by several physicians of eminent and various talents, that, excepting what relates to the views of *medical policy*, but little new matter of remarkable interest could be expected; at least, not from the physicians who had, but a very short time previously, published the results of their experience and reflections. One of these Reports is by Dr. GRATTAN, and has been already noticed in this Journal; another is by Dr. O'BRIEN, whose views of the nature and treatment of fever have also been presented to our readers. Two of them are the productions of physicians —Dr. ROBERT REID and Dr. PICKELS—who now first inscribe their names amongst those of men whose writings will be hereafter referred to, as the sources of information of sterling value respecting the epidemy which has of late contributed so much to the desolation of Ireland. The Report of Dr. Pickels relates to the South Fever-Asylum of Cork, and is chiefly of a statistical character; which is, however, illustrated by corollary observations on the relative value of different modes of treatment in the malady above alluded to. The chief intentions of Dr. Reid, in his paper, are to show how far some of the most important points in the etiology of fever may be explained by the principles which he had already advanced, in an Essay published with his Treatise on Tetanus and Hydrophobia. Although Dr. Reid does not attempt to form a general theory of fever,—(the effecting of which cannot, rationally, be expected in the present state of physiology,)—and has mingled conjectures with his precise inferences, his notions are worthy of attentive consideration: as they will, however, be noticed in the Proëmium to the next volume of this Journal, we shall not examine them in a particular manner in this place.

Taking the rest of the papers in this volume in the order in which they occur, the first we have to notice is one giving an account of "*a Case of Hæmorrhage, supposed to be from the Spleen, in consequence of Injury done to that Organ.* By Wm. Harrison, M.D. Licentiate of the King's and Queen's College of Physicians; late Surgeon of the 36th Foot."

The patient was a serjeant in the 5th Dragoon Guards, who had for many years been employed as a rough-rider in that regiment, "a tall, thin subject, with considerable muscular strength; aged 50; fair complexion, with rather florid cheeks; had accustomed himself to spirituous and porter drinking."—"He was riding a horse which he had in training, at the same time leading another by the bridle; the latter started, and dragged him off his seat, when he fell with violence between them. He was then very much hurt at the region of the spleen, and had not been able since to follow his occupation, but suffered much with pain and soreness, with a scalding at the scrobiculus cordis." Ten days after this, "I was frequently called on during the day," Dr. Harrison says, "but did not see him till seven o'clock in the evening, when I found him in a very helpless state; pulse scarcely perceptible; face and lips pale; voice and lips sunk, and articulation so indistinct as to be unintelligible; had passed a very great quantity of dark-coloured blood, mixed with other fluids, by the mouth and rectum; there were a wash-hand bason and two jugs, each containing above two quarts of this fluid, with a trifling deposit of sediment, resembling the grounds of coffee: when this fluid was made to trickle down the side of the bason, it presented the red arterial colour. I then understood there had been much more passed, which they found necessary to throw away, on account of the fetor, but it contained little feces." No part of the fluid had coagulated, and there was a small quantity of cream-coloured fluid floating on it in one of the jugs. There was no coughing at the time of its passing by the mouth. "On pressing the abdomen with my hand," Dr. Harrison says, "he did not complain of pain at any part." The patient had been seen by a medical man in the course of the day, who had prescribed some liquid medicine which was said to have had "the effect of quieting the stomach." Dr. Harrison "directed him to continue his medicine; his drink to be quite cold; to have cold air admitted freely into the room, and alum-whey given him for drink."

No recurrence of the sanguineous discharge had happened by the ensuing morning; when, on examination of the abdomen, he complained of pain about the left lumbar region. An oily enema was ordered; but, from the patient fearing that it would produce another flow of blood, the administration was neglected until Dr. Harrison's visit in the evening. "He had three evacuations in consequence of the enema, in large quantity, blended with a small quantity of feces: he now again was very languid. The following day, he said he had rather a comfortable night: I now directed sulphuric acid to be given him in mucilaginous drinks."

On the next day, it was found that he had had two stools, which, on examination, were "very dark-coloured, and exceedingly fetid, diluted largely with the same fluid, and in large quantity."—"On the third evening," the author continues, "I found the heat of body much increased; pulse somewhat hard, and conveying to the finger the catgut feel, with a double stroke, the lesser quickly following the greater, (*pulsus dicrotus* of Solano.) He complained of much pain and tenderness in the splenic region, and the abdomen appeared very tense. I now considered that, if a return of hemorrhage occurred to any amount, he, in all probability, could not survive long; and perhaps I may be considered rash in determining to abstract blood by the arm, which was done to the amount of twelve ounces, *ad deliquum animi.* Cold perspiration supervened, which not a little alarmed me: however, a comfortable, calm sleep continued during that night, and the next day he expressed himself much easier. Since this period nothing very particular occurred, but he gradually arrived to a complete state of convalescence, and is now following his occupation in very good health."

The author remarks respecting this case, that "it appears not easy to determine, with any degree of certainty, whether the blood made its way in such quantity from the spleen, by the vasa brevia, into the stomach; or that an enlargement of the vessels connected to the intestines followed, and that this very large evacuation of blood took place, which afterwards became increased by a flow from the surface of the alimentary canal, holding in suspension feculent matter."

The etiology of cases of this sort is not at all well understood. The most prevalent notion seems to be, that the sanguineous fluid is furnished immediately by the mucous membrane of the intestinal tube; but there are many facts which seem to oppose this notion. In an analogous case related by Mr. Cooke, in one of the Numbers of the Edinburgh Medical

Journal, no adequate disease of the stomach and intestines was perceptible after death ; but the liver and spleen were both in a very morbid state. We have seen two analogous cases (the blood evacuated being florid, not like venous blood, as in what is ordinarily called melæna,) occur without any evident cause, or any external injury, in both of which there was unusual tenderness and fulness in the region of the spleen ; and we have witnessed a similar instance, with similar symptoms, which happened five days after a severe concussion of the head. They all differed remarkably from ordinary melæna, especially in not being accompanied with the excessive depression of spirits, and dread of imminent death, which so constantly characterize common melæna.

The next paper is by Dr. Robinson, and gives an account of some cases of small-pox, and two of a modified form of that disease, which occurred at a charity-school at Dublin. The interesting facts of the report are these: A girl, who had not undergone any kind of pox, sickened, on the 16th of July, with what at length became confluent and well-marked small-pox. Another girl in the same school, who slept in the same room or ward, "*who had been vaccinated*," was attacked with fever on the 3d of August, and had what would be termed by Dr. Thomson *varioloid disease*. Another girl sickened on the 5th of August, and experienced a similar affection. On the 6th of August, another girl, who had undergone neither small-pox nor cow-pox, was attacked with fever, and had small-pox. The rest of the children of the school,—thirty of whom had been vaccinated, and were, for the sake of the experiment, inoculated with matter taken from the last patient with small-pox,—escaped the influence of the infection. If Dr. Robinson should publish more of such cases, we venture to suggest to him that they should be narrated in a more particular manner. It is not satisfactory to be informed that a girl "had been vaccinated ;" nor is his description of the eruption, in the cases of modified disease, sufficiently minute and perspicuous. In one of the cases, no mention whatever is made of the appearance of the cicatrices left by the pocks.

As the observation of such cases in Dublin seems to be novel, although so common in England and Scotland, we shall give Dr. Robinson's account of them in detail.

"E. Connor, *who had been vaccinated*, on the 3d of August was attacked with fever. 4th. Fever continued. 5th. Eruption appeared. 6th. Febrile symptoms subsiding. 7th. Eruption vesicular, not numerous, distinct, containing lymph ; pulse natural. 8th. Vesicles of a pearl colour, some changing to yellow. 9th. Incrustation commenced. 10th. Pustules rapidly drying on the face; some remain on the limbs; convalescent, and walking about. 11th. Some pustules still on the feet. 12th. Incrustation extended to all the pustules. 13th. Crusts of a yellow colour; where they have fallen off, the skin appears irregularly *raised* in the centre of the spot which they had covered.

"M. Montgomery, *who had been vaccinated*, on the 5th of August was attacked with fever; in the evening an eruption, similar to that on Connor, appeared. 6th. Vesicles forming, not numerous, distinct, and containing lymph; fever continues, but is not oppressive. 7th. Fever more excited. 8th. Vesicles of a pearl colour; fever subsided. 9th. Nearly as yesterday. 10th. As yesterday. 11th. Vesicles changed to yellow; incrustation commenced. 12th. Some pustules remain on the face and arms. 13th. Some of the crusts are yellow, some brownish, shining, and of a horny appearance, about a line in diameter."

After the remarks we have made on this account, it is due from us to Dr. Robinson to observe, that it was published previously to the late discussion of questions which have made more precise descriptions appear so essentially interesting in the history of diseases of this kind.

The ensuing paper gives an account of "*a Case of Idiopathic Emphysema*. By Richard Stanley Ireland, M.D."

The patient was nine months old. He had suffered severe pneumonia, which was succeeded by a troublesome cough, after a severe fit of which the emphysema appeared, at first above the clavicles, and at length about the whole of the neck and head. The pulse became small, quick, and irregular. "Great distress was apparent in the child's countenance; the face became pale, and, towards the end of life, of a livid colour; and a cold sweat broke out over the body." The child was bled every day by means of leeches, by which measure the distress in breathing was temporarily diminished. Purgatives and glysters were also administered, with apparent benefit. On the fourth day, the difficulty of breathing became extreme, and it was proposed to make punctures in the tumefied parts; but this measure was obstinately opposed by the mother of the patient. Death took place on the ensuing day. Examination of the body was not permitted.

Dr. Ireland refers to some analogous cases; but he does not appear to be acquainted with the observations of Dr. Laennec on this affection, which is one of the diseases of the thoracic organs which that physician has the merit of first describing, in its origin, in a satisfactory manner.

"*On Affections of the Cranial Brain, occurring in Infants*," is the title of the next paper. This is the Essay referred to by Dr. Whitlock Nicholl, in his "Elements of Pathology," as a development of views which he had but slightly sketched in the work just mentioned. It is an excellent and highly valuable memoir, and should be perused attentively by every medical practitioner. Since it first occurred to our notice, we have witnessed several cases which have presented proofs of the propriety of the author's views, and of the truth and minute accuracy of his distinctions, as well as of the particular success attendant on the practical precepts they have indicated.

Dr. Nicholl commences his dissertation with some appropriate critical remarks on the disposition which exists amongst medical men,—more extensively and more commonly, perhaps, than most of us are inclined to acknowledge,—to confine their regards to *consequences* of disease, and, with respect to morbid states of the brain of infants, to that which is often only a very remote consequence of what should engage our express attention—*hydrencephalus*. Dr. Nicholl then proceeds to develop his views of certain states of the brain of infants, which are distinguishable by observation of their phenomena during life, and which should, respectively, become the objects of peculiar modes of treatment. He first observes that—

"There is a state or condition of the cranial brain in infants, which may be called a state of irritation, an irritated state, or, in a word, erethism. What this peculiar condition of the cerebral structure is, I cannot explain: it is a state distinct from that which is called inflammation, for it may exist without any perceptible increase of the quantity of blood received by the cerebral blood-vessels. It is a state in which inordinate effects arise in the cerebral structure from ordinary impressions upon different parts of the nervous system. In its perfect form, and under a high degree of it, it is a highly sensitive condition of the cranial brain,—a condition the very reverse of that state under which sleep occurs. Under such a condition of the cerebral substance, the child is wakeful, restless, attentive to every sound and to every object of sight; irritable in temper; the retina is highly sensible to light, the pupil generally more or less contracted; the limbs much in action; the head suddenly moved about, or shaken from side to side; and a degree of animation and a quickness of observation are present, much beyond what is commonly seen in children of the same age: so that, although a morbid condition of the cranial brain be present, the infant may be considered as perfectly healthy, on account of its being lively and sensible to the most trifling impression. By an attentive observer, other symptoms may be noticed: the child starts when asleep; when awake, a sudden frown passes over the forehead, and then disappears; the eyes are sometimes closed irregularly, or alternately; a winking of one eye, or of both eyes, may sometimes be detected; the hand is often carelessly passed over the forehead, or over the side of the head; the child cries without any evident cause, at other times it shrieks; the fists are clenched, the fore-arms bent upwards on the arms. Such a state of the cranial brain constitutes the simple form of what may be termed ***sensitive erethism.***

"There is another form of erethism of the cranial brain in infants, in which there is a great want of animation, the child being dull, yet fretful if roused; the head, perhaps, being suffered to droop, or being reclined on either side; there being an absence of sleep, a state that can scarcely be called waking; an indisposition to move; an indifference towards all objects; a general pallor and chilliness of the body; a dull state of the eyes, a rolling or turning-up of the eyes; a plaintive moan, or occasional shriek; the child awaking from sleep with a note expressive of pain or of displeasure; a wrinkled state of the integuments of the forehead; the hands raised towards the head; the pupil more or less contracted; there being apparently no notice taken of any object of sight; the body and lower extremities being, perhaps, extended; the head thrown backwards. This form of erethism may be distinguished by the term *torpid erethism.*"

The term *torpid erethism* is not, we think, well chosen; but that there exists such a peculiar state as the author describes, we are well assured by experience, and we think it is most forcibly characterized by the observation that the child is "dull, yet fretful if roused." The patient will lie in a state of apparent sleep, for hours even, if left in perfect quietude; but the slightest exciting cause is sufficient to produce great agitation and fretfulness: it is often impossible, with the most gentle manners and soothing and patient conduct, to feel the pulse, until after a long period of agitation, when the object is defeated by the great disturbance of the circulation that has been produced. The child will lie as if it were asleep, (excepting that the eye-lids are not quite closed,) but, after the most gentle approach to it, the slightest touch of the fingers to its wrist will rouse it to a state of agitation of the hands, and perhaps of the whole body, that cannot be appeased, whatever may be the age of the infant, by any soothing intreaties. It is but very rarely possible to inspect the tongue, except in a very imperfect manner, as it may be seen when the child cries; nor can it hardly ever be ascertained whether there is tenderness in the belly; but, as far as we have been able to observe, the tongue is ordinarily dry, somewhat smooth, and of a brownish colour.

Previously to a consideration of the evident exciting causes of erethism of the brain of infants, regarded generally, Dr. Nicholl adverts to a state of that organ consisting in some peculiar original structure, which predisposes to it. Sufficiently close and deep investigation must,—as is shown in a modern work, in which this point is first discussed in a thorough and determined manner,—indeed, lead us to this unknown term of causation in most diseases.

The exciting causes of this erethism enumerated by Dr. Nicholl, are most of the common exciting causes of disease; but especially undue impressions on the expansions (or, as Dr. Nicholl terms them, the anti-cerebral extremities,) of the nerves spread out in the substance of the liver and the alimentary tubes; and an increase of the quantity of blood circulating through the vessels of the brain.

"As, on the one hand," he says, "we find that an increase of the quantity of blood that circulates through the cerebral blood-vessels tends to the production of erethism of the cranial brain, so do we find that erethism of the cranial brain may cause the cerebral arteries to receive more than their due share of blood. So that, after erethism of the cranial brain has existed for an uncertain time, an increase of the quantity of blood that circulates through the cerebral blood-vessels may be superadded to the erethismal state. The combination of a great increase of the quantity of blood that circulates through the cerebral blood-vessels with erethism of the cranial brain, constitutes that state which is called inflammation.

"Inflammation of the cranial brain in infants is, then, a state which includes in itself erethism of that substance; but erethism of the cranial brain in infants may exist independently of any perceptible increase of the quantity of blood that circulates through the cerebral blood-vessels. When erethism of the cranial brain exists in combination with a great increase of the blood that circulates through the cerebral blood-vessels, such erethism may either precede or be superadded to that increase.

"A highly sensitive form of erethism of the cranial brain in infants, coupled with a great increase of the quantity of blood that circulates through the cerebral blood-vessels, constitutes what is called active, or acute, inflammation of the cerebral substance. A less sensitive form of erethism of that substance, combined with a more moderate increase of the quantity of that blood, constitutes, probably, what had been termed a sub-acute form of inflammation of the cranial brain; and a moderate increase of the quantity of that blood, joined to the more torpid form of erethism, may, possibly, constitute the chronic form of cerebral inflammation."

After a description of the symptoms of inflammation of the brain, Dr. Nicholl speaks of *simple plethora* of the cerebral blood-vessels of the brain of infants; the causes of which—as general increase of the quantity of the blood; impediment to its return from the head; impediment to its distribution to other parts of the body; diminished tone, and consequently diminished resistance to its entrance in them, of the blood-vessels of the brain; increased temperature of the head, and "certain impressions on the anti-cerebral extremities of nerves, as on those in the liver, in the alimentary canal, in the gums,"—are passed in review. That fullness which depends on simple obstruction to the course of the blood from the veins, he terms "congestion in the cranial brain." He then adds that—

"The quantity of fluid poured out by exhalants in different parts of the body, is very much influenced by the state of the nerves in the vicinity of such exhalants. Whatever produces such a state of those nerves as is expressed, rather than explained, by the term *irritation*, disposes the exhalants in their neighbourhood to pour out an extra quantity of fluid. In like manner, we find that erethism of the cranial brain disposes the cerebral exhalants to pour out an increased quantity of fluid into the cavities of that substance.

"In some cases, when an impression on distant anti-cerebral extremities of nerves, (such as those which are spread out in the liver, or throughout the alimentary canal,) produces such an effect at the cerebral termination of those nerves,—*i. e.* on the cranial brain,—as causes the cerebral exhalants to pour out an increased quantity of fluid, we find that there are scarcely any symptoms present which denote the existence of erethism of the cranial brain; the symptoms which arise being chiefly, if not entirely, such as may be attributed to the presence of a collection of fluid in the ventricles of the cerebral structure. And, in some of these cases, the increase of the quantity effused by the cerebral exhalants is so moderate, that a considerable length of time may elapse ere such a collection is formed as may give rise to marked and decided symptoms. In such cases, it is very probable that a collection of fluid in the ventricles may take place before any suspicion is entertained of diseased action going on in the cranial brain, or of the existence of any source of irritation in any of the contents of the abdomen."*

After some further discussion of the means of accumulation of serous fluids in the brain, Dr. Nicholl observes, in a summary way, that—

"In inflammation of the cranial brain in infants, there exists a two-fold cause of an undue flow of fluid from the cerebral exhalants. In the first place, such a state includes erethism, which, as I have already stated, disposes the cerebral exhalants to pour out an increased quantity of fluid. In the second place, the cerebral arteries, during inflammation of the cranial brain, receive an increased quantity of fluid, and, consequently, an increased quantity must pass by the terminations of those arteries, of which terminations the cerebral exhalants form a part.

"A faulty state of the exhalants of the cranial brain may dispose them to give passage to a preternatural quantity of fluid.

"As the fluid usually poured out by the cerebral exhalants is, under a due and healthy action of the cranial brain and of its vessels, removed in proportion as it is effused, by which removal an accumulation of that fluid is prevented, it follows that, if the vessels whose office it is to remove that fluid perform that office imperfectly, an accumulation

* In cases of this kind, instead of a contracted pupil, we may find the pupil of a natural size, or it may ultimately be dilated.

of fluid in the cavities of the cranial brain will take place, although the exhalants pour out only a due and natural quantity."

The next object of the author is to distinguish the states of simple plethora and congestion.

"In simple plethora of the cerebral blood-vessels, there is increased heat of the head; a full eye; a redness of the countenance, together with a want of animation; a heavy, listless state; indisposition to move the head; uneasy sensation of fullness in the head, causing the child to seek support for the head; giddiness; sometimes shaking of the head; uneasy respiration; the pupil of a natural size, or perhaps rather dilated; sickness; loss of appetite; heat and dryness of the mouth and skin; an inactive state of the bowels; a sensibility to impressions, but a heedlessness of them; a turgid state of the vessels of the head; the pulse not much accelerated, full, perhaps oppressed.

"In congestion in the cranial brain, there is a general coldness of the body; the child throws the head backward, or leans it on either side; the child lies in a comatose state; the eyes prominent and fixed, the pupils fully dilated, not contracting on exposure to strong light; stiffness of the body, and of the limbs; irregular actions of the muscles of the face and eyes; a more or less entire absence of vision; a more or less complete state of insensibility to all impressions; torpid state of the bowels; scanty or suppressed secretion of urine; pulse, for the most part, slow, and oppressed."

Nearly similar (too nearly alike to be distinguished in many cases,) to the symptoms of congestion, are those of undue accumulation of fluid in the brain.

The author concludes his observations on the points above noticed, with stating that "erethism, plethora, inflammation, and congestion, may severally so affect the cranial brain as to bring on death without having previously produced effusion into the ventricles; or these states may severally subside without giving rise to such effusion; or these states may severally continue, effusion being also present; or these states may cease, and effusion may remain as a consequence."

Besides the conditions above designated, Dr. Nicholl says "there is a state of the cranial brain which may be termed torpor, or insensibility; a state characterized by a general insensibility to impressions of every kind, and by all those other symptoms which have been enumerated as the consequences of congestion in the cranial brain, or of effusion into the ventricles." And, he adds,

"Torpor of the cranial brain may arise from whatever causes that substance to be unduly compressed between its unyielding parietes externally, and its own blood-vessels internally. Thus it arises from a plethoric state of the cerebral blood-vessels; from congestion in the cranial brain; from a collection of fluid in the ventricles,* (arising

* During a state of torpor of the cranial brain, do those vessels whose office it from any of the causes already mentioned, whether erethism, simple plethora, inflammation, congestion, or a faulty state of the cerebral exhalants, or of the cerebral absorbents;) from effusion of blood; from collections of pus; from tumors; from thickening or indentation of the cranium, and the like. It may arise from concussion of the cranial brain; it may be the consequence of a preceding high degree of the erethismal state of the cranial brain; it may arise from some peculiar alteration of the cerebral structure, or from loss of a part of the cerebral substance; or it may be connected with, and dependant upon, original formation. It may arise from a loaded state of the alimentary canal, or from some morbid condition of the passage, especially of the internal membrane of the canal; from worms in that canal; from disease of the mesenteric glands; from congestion of bile or of blood in the liver, or from some other faulty state of that viscus. It may arise from loss of blood; from extreme cold; from diminished action of the heart; from disordered action of the lungs; from vitiated atmosphere; from respiring carbonic acid gas; from intoxication; from sedatives, externally applied or inwardly administered; from poisonous substances, &c."

The different states of the brain of infants, as considered by Dr. Nicholl, may then be arranged under the heads of Erethism, (sensitive and torpid ;) Simple Plethora; Inflammation; Congestion; Collection of Fluid in the Ventricles; and Torpor.

"I think that I am correct," Dr. Nicholl says, "in stating that each of these conditions of the cranial brain may exist as a distinct affection; and that, in the pure, uncombined form of each of these states, each condition has distinguishing characters by which its presence is denoted. But, when we review these various conditions, and consider how they may be blended; when we look over the list of causes from which they may severally arise, and see how many of the causes of each condition may exist at the same moment; and when we take into the account the influence which the various modes of treatment have upon disease, how much they alter its features, and how much they modify and interrupt the train of its symptoms; we shall be prepared to meet with appearances very different from those which are traced upon paper, and we shall not be surprised at being called upon to treat cases in which there is an assemblage of symptoms that baffle all our attempts at classification, and that defy all nosological distinctions."

Several of the diagnostic phenomena of those conditions are, in the next place, discussed in a particular manner: but we cannot follow the author on this occasion; and we consider, too, that the principal inferences have been already indicated in our transcriptions.

---

is to remove the fluid usually effused by the cerebral exhalants, ever partake so far of the general torpor as to perform that office imperfectly, or to cease entirely to perform it? If they do, torpor of the cerebral substance may cause a collection of fluid in the ventricles, although the cerebral exhalants pour out only a natural and due quantity of fluid.

Having discussed in this manner the subjects of his dissertation, the author takes a somewhat more general view of their relations in the economy.

"Whatever," he says, "may be the form under which a diseased condition of the cranial brain in infants manifests itself, certain facts must be borne in mind, namely,—That, although the cranial brain appears to be the seat of disease, yet that the primary cause of the morbid condition of that substance may exist in some part far distant from the cranial brain; as, for instance, in the neighbourhood of some anti-cerebral extremities of nerves, which are spread out in the liver or in the alimentary canal; and that, as long as such cause remains and continues to operate, it is absurd to expect to get rid of the affection of the cranial brain. Secondly, that, although such cause be removed, or have ceased to operate, yet, if it have existed for a considerable length of time or have been very powerful, it may have induced a state of the cranial brain, which may remain after such cause is removed. Thirdly, that either of the states of the cranial brain which I have mentioned may arise from various, and from very different, causes. Fourthly, that the different states require different modes of treatment. And lastly, that either of these states may induce, or may pass into, a different state: thus, simple plethora may induce erethism, or may be induced by this last state, and, in either case, inflammation will be present; or plethora may bring on torpor; torpor may succeed to erethism, to plethora, to inflammation, to congestion;* sensitive erethism may pass into torpid erethism; in inflammation, the plethoric state of the cerebral blood-vessels may subside, and erethism may alone remain."

The most common of these affections, is that which Dr. Nicholl has endeavoured to express by the term *erethism;* the other conditions being, in many cases, consequences of that state. The predisposition to this state is often manifest for a long time before any disease is supposed, by the parents of the patient at least, to exist. The most common attendants of it are, a precocity of intellect,—as we remarked in our review of Dr. Cheyne's Essay on Hydrencephalus;—a general appearance of the child ordinarily intended to be expressed by the term *scrofulous;* "either a highly sensitive state of the retina, with a contracted pupil, or a general langour of expression, with dilated pupil;" great irritability of temper; and an extraordinary degree of liveliness and animation.

The influence of improper diet, as an exciting cause, is somewhat extensively discussed by the author. When noticing the agency of disorder in the alimentary canal on the brain, he remarks, "we must not forget that a morbid condition of the cranial brain influences the secreting action of the liver, either increasing that action, or altering it, or lessening it: we must,

* May not torpor of the cranial brain induce congestion in that substance?

therefore, be very cautious to distinguish between cause and effect in these cases; or else it may happen that, while we are endeavouring to improve the action of the liver, in order that we may remedy the condition of the cranial brain, we ought to be endeavouring to improve the condition of the latter, in order that we may produce improved action of the liver. The converse of this supposed case may also occur. The same observations are applicable to imperfect or faulty action of the alimentary canal, as connected with the state of the cranial brain."

We are convinced that the morbid condition of the alimentary canal is much more frequently a consequence of the disorder of the brain, than some late writers on this subject would have us believe: the black and otherwise disordered feces very frequently do not appear until the cerebral affection has evidently existed for some time. This is particularly remarkable in cases originating from blows on the head, which is a frequent origin of the disease amongst poor children, who are so commonly nursed by very young girls, and are thus exposed to such accidents. Dr. Harrison has mentioned to us several interesting facts related to this point, which he has witnessed in his practice in the treatment of spinal diseases. The dark colour and otherwise disordered state of the feces, (which very commonly present a remarkable odour, that he says was pretty well designated by the mother of one of his patients, when she compared it to that of *the washings of a gun*,) which so commonly accompany a certain extent of spinal deformity, ordinarily disappears almost immediately, (without the use of medicine,) often on the next day, after a certain degree of removal of the deformity, even when this has been effected suddenly,—that is to say, in an hour or so. The ammoniacal state of the urine, which is sometimes, also, a symptom, disappears with equal rapidity.

The first interesting remarks by Dr. Nicholl on the treatment of the affections under consideration, are the following:

"In children of a very languid habit, conjoined with that state which shews a tendency to erethism, we must be cautious how we administer calomel too frequently, with a view to excite due action of the liver. Perhaps calomel is, in many cases, given in repeated doses for a considerable length of time, for the express purpose of removing an appearance of the stools which is solely kept up by the calomel itself: the appearance to which I particularly allude, is the green hue, resembling that of stewed spinach,—an appearance which is frequently represented as being characteristic of such a condition of the cranial brain as terminates in hydrencephalus. Calomel is given to promote secretion of bile; it is also given where that secretion is too abundant: the evil is, that it is given in a similar manner as a remedy in these opposite states. There is no absurdity, no inconsistency, in exhibiting it as a remedy in both these states, if we adopt the mode of giving it to the particular state. For instance, if the object be to promote the secretion of bile, we should give small doses in repeated succession, so as to produce the specific effects of mercury on the liver: if we want to remove from the alimentary canal an inordinate quantity of bile which has flowed into it, we should give one powerful dose of calomel, so as to produce its effects as a strong purgative on the intestines. But, in very languid habits,—in habits which show a tendency to scrofula, to erethism of the cranial brain,—if we want to promote a more copious secretion of bile, it will be more prudent to endeavour to attain that object by employing other remedies,—such as nitro-muriatic acid, internally and externally, or small doses of sulphat of potash, with extract of taraxacum, with a little aloetic wine; or, if we find it necessary to employ mercury to promote the action of the liver, the mercurial pill, the grey oxyde of mercury, the hydrargyrus cum cretâ, are less objectionable forms of that mineral. These several means may be assisted by warm salt-baths, by friction with salt, and by clothing the child in flannel."

We are, perhaps, more averse to the continued use of calomel, or mercury in any form, than Dr. Nicholl, in the kind of patients he describes: it is, however, very fortunately, often found that one large dose of calomel will produce a due appearance of bile in the feces, without giving rise to the ill consequences which result from establishing the action of the mineral in the system generally. It is true that the green colour of the stools, with the absence of yellow bile from them, will frequently return after a few days, if other and effectual means be not employed to preserve the action of the liver; but such measures may often be found in less objectionable medicines than mercury. We always, at least, try the practice just designated; and, when we resort to a continued course of mercury, in case of the failure of that practice, it is with the suspicious caution with which a man follows a villainous guide, at night, in a strange country.

When erethism of the brain exists in a high degree, whatever may be its cause, it is necessary to use means for the immediate alleviation of it; for which purpose, Dr. Nicholl says he knows no remedy more appropriate than the pulvis ipecacuanhæ compositus. This medicine had already been forcibly recommended for concussion of the brain, and in the early stage of, as it has been called, "*hydrencephalus*:" but we think much advantage will be derived from Dr. Nicholl's more precise indications of the states in which it is expressly proper, which are founded on the important distinction between erethism and inflammation. With this opiate, antimonial powder, especially in what Dr. Nicholl calls *sub-acute inflammation*, cold lotions to the head, quietude, nitre and acetate of potash, mild glysters, and a "tepid bath of moderate temperature," may be conjoined. "In children of a full habit," Dr. Nicholls adds, "if the symptoms of erethism of the cranial brain run high, leeches may be applied to the temples. But, in that form of affection which I have endeavoured to describe under the term *torpid erethism*, where there is great general pallor and coldness, insensibility, and contracted pupil, the abstraction of blood will be a dangerous experiment, and may hurry our little patient out of the world. In such cases, we must endeavour to combat the affection by the means already pointed out; and, in addition to these, the head may be blistered."

In all cases of erethism, Dr. Nicholl takes care to observe, "we must ever be on the watch for the supervention of a plethoric state of the cerebral blood-vessels, which, combined with the erethismal state, constitutes inflammation." If this occur, and is removed by the proper measures, an erethismal state may yet remain; when "it will be proper," Dr. Nicholl adds, "to give the pulvis ipecac. comp. in liberal doses, combined with James's powder and nitre, with small doses also of calomel, or of the hydrargyrus cum creta. For, if the erethismal state of the brain be not allayed, the child will continue restless, wakeful, and irritable; and we may expect that the plethoric state of the cerebral blood-vessels will, sooner or later, return, and that, in this way, the child may at length be worn out."

A case is related by Dr. Nicholl, in a subsequent part of this volume of Transactions, that exemplifies very forcibly the most important of his general observations respecting erethism of the brain of infants; and, as we have cited so extensively from his dissertation on this subject, or, in other words, have pretended to give a complete abstract of it, we consider ourselves obliged to cite also so striking an illustration of his views. The account of the case will not admit of abridgment, and is as follows:

"Mr. Acton, a very intelligent surgeon of this town, (Ludlow,) has an infant daughter, who is between eight and nine weeks old; she was, from her birth, lively, very wakeful, scarcely ever sleeping during the day; highly sensible to impressions: when she was scarcely six weeks old, she awoke as with a hesitation of breathing, and the muscles of the face were convulsed. She became still more restless, and was very fretful. Nothing amiss had ever been noticed in the character of her stools. She was suckled by her mother, a very healthy young woman. Her father gave her a dose of calomel, and put her into a warm bath: the stool which succeeded to the exhibition of the mercurial purgative, was perfectly healthy. After this I saw the child: it started when the door was opened, or when a chair was hastily moved, or when any one coughed, or if any part of its body was touched. It cried very much and very loudly, and was only appeased, and that momentarily, by being placed in a sitting posture, by being carried about, or by being put to the breast. The pupils were of a natural size; there was no vomiting; no heat of skin, no heat of the head, no flushings of the cheeks, no increased throbbings of the arteries of the neck and head. When this highly sensitive and wakeful state had continued for several hours, the child became gradually more heedless of noises, until at length it ceased to notice them; the crying then subsided, and the child bore a horizontal position. In this state, the eye appeared as if insensible to the light of a candle, the pupil, which was rather enlarged, vibrating, as it were, between contraction and dilatation when strong light was thrown on the eye; the fore-arm bent on the arm; the fingers clenched; the thumb laid flat across the palm; the upper extremities, in this state, raised, in constant motion; the head sometimes moved about, but not much so: the lower extremities sometimes suddenly drawn up; the lips moving; no moaning; occasional rolling of the eyes; the eyes fully open; not a moment in which some muscles were not in quick action; the body bent backwards. When this state had continued for four or five hours, sleep came on, out of which the child awoke, and appearing in its usual state; its arms pliant; its hands open. Then came on the fretful, crying, restless state; then the torpid restless state, during which the muscles were in constant action; the fore-arm bent; the fingers clenched as before; then sleep; after which, apparent recovery. And thus did the sensitive erethismal state, followed by torpid erethism, by sleep, by recovery again, repeatedly run its course. The brain, after the highly sensitive state had been long kept up, gradually assuming a state approaching more and more to torpor, until its actions were at rest, and then was sleep present; but, after a short rest, the brain *awoke* to its original state. It was remarked that, when the sensitive state of the brain recurred, the bowels were relaxed, notwithstanding the use of opium; the eyes were suffused; the child sneezed, and had an increased quantity of moisture in the nostrils and of saliva from the mouth: when the sensitive state declined, the bowels were no longer relaxed; the coryza disappeared, secretion having been increased by the erethismal state. At one period, during the torpid erethismal state, there was complete opisthotonos to a great extent, so that the spinal brain was affected also with the erethismal condition.

" The head, first of all, was blistered: during the state of opisthotonos, the whole of the spine was blistered. The application of the blister to the spine appeared to give much relief, especially by its first operation; afterwards it was thought to irritate too much. A grain and a half of Dover's powder, was the remedy always resorted to: if given during the highly sensitive state, it allayed the irritation; and, when given during the more torpid state, sleep gradually came on. In one instance, the fretful and the sensitive state, and the more torpid state, occupied two nights and the intervening day, during the whole of which there was scarcely any sleep,—none for a longer period than a few minutes; then sleep came on, which lasted several hours. The

Dover's powder generally quieted the child in three or four hours; a tea-spoon full of syrup of poppies had no effect at any time. Musk had no good effect. The muscular actions generally came on at night. I gave decoction of bark in one of the intervals, a tea-spoon full every hour: I thought that this, combined with the p. ipecac. c., had a slight good effect; but it was not followed up, as Mr. A. thought that the child was in pain after taking it. James's powder made it sick. After the child had continued about a fortnight in this state, the train of symptoms being repeated every day, or every two days, it has continued for the last fortnight without any marked symptom of disease, being better than it has been since its birth; yet there is still an absence of sleep during the day; so that I suspect that there exists some congenital formation of the cerebral structure, which is incompatible with the long duration of health, and perhaps with that of life. The case, as yet, has been a well-marked one of pure erethism, unmixed with the slightest perceptible alteration in the state of the blood-vessels, and alternating with a more torpid state, which is the consequence of the previous highly sensitive state."

The paper we have to notice in the next place gives the history of a "*Case of Amputation at the Hip-joint, for the removal of an Osteo-sarcomatous Tumor*. By Richard Carmichael, Esq. surgeon of the Richmond Hospital, House of Industry, &c. &c."

The subject of this operation was a "delicate-looking girl, about nineteen years of age, of a fair, but now sallow, complexion." The tumor was of the species termed *fungous exostosis* by Mr. Astley Cooper, and extended "from just below the knee, as far up the thigh as barely to leave room for the formation of the flaps; and the circumference of the limb, where it was most prominent, measured twenty-seven inches." The disease had been of twelve months' duration. The operation appears to have been performed with great dexterity and rapidity, and the patient appeared to be doing well until the fourth day after it, when she became restless and uneasy, and soon manifested signs of the existence of great constitutional irritation, which increased until the sixth day, when she died. The femoral artery was not tied, as a first step of the operation: the artery was commanded in the groin, by an assistant. "The entire line of the wound," of the stump, had united by the third day. Some tension was, however, now experienced in the part: the ligatures were removed on the next day, when the stump "appeared full and distended," and "it was suspected that an accumulation of serum had occurred;" but the patient would not permit a probe or director to be passed through the new-formed adhesions, that it might be ascertained if such were the case. On the fourth day, the stump was "greatly swoln, as if distended by some fluid; but, on introducing a director through the new-formed cicatrix, there did not come any more than half an ounce of thin ichor. Cold applications had been applied, and were continued. About half an ounce more of ichor came away on the next day. The following is the account of the appearances of the stump after death:

"Upon examining the stump, which was done by separating the flaps that had united through their entire extent externally, a large spongy mass, not unlike a piece of unhealthy lung, presented itself. On cutting into it, we ascertained that it was the divided muscles which were thus strangely altered in so very short a space of time; for during the operation they presented a healthy appearance. It was this swelling of the muscles which caused the tumefaction of the stump already mentioned; for scarcely any fluid came away previous to death, on the introduction of the probe or director.

"The little ichorous matter which did pass off, was ascertained to come from the upper part of the wound, where there was a considerable cavity, the bottom of which was formed by the acetabulum."

No morbid appearances were observed in any of the viscera.

An account of a case of *Cynanche Laryngea*, in which tracheotomy was performed, that presents several interesting circumstances, is also furnished by Mr. Carmichael. The peculiar advantages derived from removing a portion of one or two of the rings of the trachea, as recommended by Mr. LAWRENCE, were particularly manifest in this case. An attempt was once made to keep a canula in the wound, but "the excessive irritation which it occasioned, rendered its immediate removal necessary." The edges of the external wound were kept separate by two retractors covered by adhesive plaster, which were connected to each other by two strings tied behind the neck. It was deemed necessary, however, to enlarge the opening on the evening of the second day, from the great quantity of viscid mucus which accumulated in the trachea obstructing the original one. The patient recovered. The wound had healed on the fifteenth day.

Another case in which tracheotomy was performed by Mr. Carmichael, gives this surgeon an opportunity for adducing several hints, which he thinks of some import to practitioners. He was called, late at night, to perform tracheotomy on a woman who was in a state approaching suffocation from an inflammatory affection of her throat, which she had complained of for more than a month previously. It had been attended with great difficulty in swallowing as well as in breathing, and had been treated by local and general blood-letting, vesicatories, and other measures, under the direction of Dr. MILLS.

On examining the fauces, Mr. Carmichael could not perceive any swelling of the tonsils, or of the posterior part of the pharynx. "The entire front of the neck was, however, so much swoln, that it was impossible to feel the trachea; and even the larynx itself was scarcely distinguishable." Considerable hemorrhage, and various other means of difficulty, in the thickness of the parts over the trachea, attended the operation; and, from the blood getting into that canal, Mr. Carmichael did not attempt to enlarge the wound (a simple incision) in the trachea, or to introduce a canula, for fear of renewing the hemorrhage. She was left "totally free from convulsive cough or stridulous breathing; some air passing through the wound, but the greater part by the glottis." She continued during the next day in a tranquil state; but in the evening the wound had nearly closed, and little advantage was obtained by endeavours to enlarge it by means of a probe. No air passed by the wound on the next morning, and the difficulty of breathing was extremely great. An attempt was made to enlarge the opening in the trachea, "when a smart hemorrhage occurred from an artery in the upper part of the wound, which was with some difficulty secured."

Towards the evening, the swelling had subsided, and Mr. Carmichael then was able to introduce a canula without any further assistance from the knife. The canula, after a few minutes, produced but little uneasiness. But the patient on the next day became totally unable to swallow, and a gum-elastic tube excited such irritation and convulsive efforts, as soon as it reached the seat of the obstruction in the œsophagus, that the attempt to convey nutriment into the stomach by means of it was necessarily relinquished. The patient suffered acute pain from hunger, and, as she could not speak, stated by writing that she was starving. Glysters of broth, milk, and opium, were administered. She sunk rapidly, and died on the next morning, (the fifth day from that of the operation,) almost immediately after having vomited "a quantity of purulent matter."

On examining the body, "an abscess containing about six ounces of purulent matter was discovered, extending from the second or third cervical vertebræ as low as the sixth or seventh, situated between the bodies of the vertebræ and the posterior boundary of the œsophagus; the walls of the abscess were firm and unyielding. The matter which she vomited just previous to death, as well as some which was found in the trachea, must have escaped through a small opening in the most prominent part of the abscess, opposite the upper portion of the larynx, as there was no other source ascertained from which it could have been derived."—"The vertebræ, larynx, pharynx, and œsophagus, were of a natural appearance; as were also the lungs, except that there were extensive old adhesions between the pleura costalis and pulmonalis on both sides of the chest!"

"The practical lesson we derive from this case," Mr. Carmichael says, "is of the highest importance; for, if I could have been aware of the existence of the abscess, a simple puncture into it would, in all probability, have saved the patient's life."

"It is true," Mr. Carmichael adds, "that abscesses are every day met with on the back of the pharynx, obvious to the sight and touch; for these the remedy is apparent, and affords immediate relief. But my object is to draw the attention of the profession to those abscesses, which, as occurred in the cases just detailed, are situated so low as not to be visible, and are, in many instances, beyond the reach of the finger. Such cases may be indicated by the precise seat of the pain; the obstruction, in the first instance, to the passage of aliment; afterwards, as the swelling increases, to the passage of air; the slower progress of the symptoms compared to those of laryngitis; and the more rapid, when compared to those of stricture of the œsophagus, or of chronic tumors pressing upon that canal;* the obstruction to the passage of an instrument down the œsophagus, while those complaints about the fauces which might occasion obstruction, are absent; a general swelling of the anterior part of the neck almost approaching œdema, analogous to what occurs on the surface over any deep-seated abscess; and, possibly, the accession of irregular shiverings, although it was not remarked that this symptom occurred in either of the cases detailed.

"The existence of these, or one or more of these symptoms, should induce an examination with the finger; or, if the finger cannot reach the obstruction, a gum-elastic bougie or sound should be passed, and the obstruction will point out the situation of the abscess. If we are satisfied on this head, a curved trocar, somewhat similar to that recommended by Sir E. Home for puncturing the bladder through the rectum, may then be passed, the distance at which the sound met the obstruction being previously marked upon it: and here the stilet, being made to protrude, may be boldly plunged into the tumor, taking care to push it towards the central line of the bodies of the vertebræ, where no danger can arise from the puncture. If matter flows, we will save the life of the patient; but, if we are disappointed, no material injury, that I can conceive, will be the consequence of the attempt."

"If the practitioner is timorous about passing a trocar down the œsophagus, a silver catheter forced into the abscess may answer equally well, and in most hands may be found the safer instrument."

---

* These tumors are by no means unfrequent, numerous instances of which may be found in the writings of the older medical authors. Lieutaud alone details four cases of firm tumors impeding deglutition by pressing on the œsophagus.—Vide Lib. iv. Obs. 93, 95, 98, and 99.

A paper containing an account of "*a Case of Gangrene occasioned by the use of Mercury*," by Dr. GRATTAN, occurs next in order. This paper is a particularly interesting one, for several reasons: the case itself is highly so, and it is gratifying to contemplate the sentiments which prompted the publication of it. Dr. Grattan accompanies the narration by some good and appropriate considerations on the uncertainty of the agency of our remedies, and the danger we encounter of occasionally, either witnessing unfortunate results from them, when energetic, or of meriting censure for failing to employ them with due vigour in cases where, had they been thus employed, success would have crowned our efforts.

A girl, ten years of age, who had complained for some weeks previously without exhibiting any evident signs of fever, at length experienced this affection in a decided manner, and was removed to a fever-hospital.

"The child, when visited, obviously laboured under febrile excitement, and appeared to be in the last stage of disease. The head seemed to suffer most; and, from her general appearance, I was led to conclude that water either had collected in the brain, or was on the point of being effused into the ventricles. Delirium, with frequent screaming, alternated with occasional intervals of stupor or heaviness, black dry tongue, pulse seldom under 130, beating of the temples, face sometimes pale and sometimes flushed, were the most prominent symptoms. A two-grain pill of calomel was given, succeeded by a draught of the oleum ricini. Leeches were applied to the temples, and the head was shaved. The symptoms were not improved. The temporal artery was then opened, five ounces of blood were taken, and a blister applied to the occiput and nape. A pill, consisting of two grains each of calomel and ipecacuanha, was given twice, and sometimes thrice, a-day, with the exception of those days on which oil-draughts were administered; so that, by the end of the sixth day after admission, ten pills had been taken. The gums now became sore, and every alarming symptom disappeared. Copious ptyalism succeeded, accompanied with great tumefaction of the face and lips. This state had continued for four or five days, when a small vesicle was observed near the left angle of the mouth, which in a short time assumed a black colour, and in a few hours increased to the size of a sixpence. It was unaccompanied by the slightest perceptible inflammation, and occasioned no pain whatever. By the following day it was as large as a half-crown piece, and in appearance exactly resembled a superficial sore that had been dusted over with finely powdered charcoal. The salivation continued, attended with great fetor. The fermenting poultice was applied; bark and opium were administered internally. Wine was given; the sore was also occasionally washed with tincture of opium, and sprinkled with the powdered bark, but without effect. The gangrene extended itself until a part of the cheek, about two inches in breadth, was eaten away. The right angle of the mouth was now affected, the disease commencing precisely as already described. The respiration became laborious and oppressed; and death at last took place on the nineteenth day after admission, being the thirteenth day from that on which calomel had been last prescribed, and the eighth from the time when the gangrene first shewed itself.

"Whether the above was from its commencement essentially a case of hydrocephalus internus, I am by no means prepared to decide. Whether the accompanying fever was symptomatic of the primary hydrocephalic affection, or whether a low irregular fever, neglected at the commencement, and acting on an organ preternaturally disposed to derangement, might not have occasioned in the brain that particular morbid condition which the symptoms so strongly indicated, it is by no means easy to determine. From the malignant character which the fever assumed, and the marked typhoid symptoms which attended it, I am inclined to adopt the latter supposition. However, in either case, the brain being the organ affected, and under a state more or less of inflammatory excitement, the leading indication evidently was to allay that excitement, and to correct any mischief which it might have occasioned by its continuance. With this view the local abstraction of blood was resorted to; and, in order more effectually to accomplish the same object, mercury was prescribed so as to affect the system. In the course of six days twenty grains of calomel were taken; the mouth became affected, and immediately the patient appeared free from disease. So far every thing proceeded more favourably than, under such unpromising circumstances, could have been expected, and so far the propriety of the course adopted seems to be confirmed by the result. But, an unexpected train of symptoms succeeding, and terminating in a manner so unfavourable, we are naturally led to inquire into their causes. It becomes a matter of much importance to determine, whether they depended on the previous treatment, or whether they should rather be ascribed to a different origin. In the present instance, I think that the mercury, though it chiefly contributed to the cure of the primary disease, yet, acting on a system of extreme delicacy, was nevertheless the cause of the subsequent untoward event. It would appear, from the violent effects of the medicine, that there existed in this instance an unusual susceptibility of constitution, by which its ordinary influence was modified and rendered noxious; for much larger quantities of mercury are frequently exhibited to younger patients without producing salivation. Could this peculiarity have been foreseen, it is evident that the medicine should either, in the first instance, have been entirely withheld, or administered with greater caution. On the other hand, were we to anticipate in every other case a similar result, our practice would become so vacilating and timid, that a great proportion of our patients might die through our hesitation in not employing an active remedy until we had first satisfied ourselves, by cautious experiment, that it was not likely to prove injurious. A few exceptions ought not to preponderate against the great majority of well-established cases, in which a particular treatment has been safely and beneficially adopted. We do not consider the remote possibility of aneurism, or of secondary hemorrhage from the temporal artery, sufficient to contra-indicate the employment of arteriotomy in a case of violent phrenitis. Thus, also, were I again to treat a patient similarly affected, I should not hesitate to have recourse to mercury, as the medicine on which, after blood-letting, I ought chiefly to rely."

In addition to the foregoing remarks, Dr. Grattan observes that a gangrenous affection, similar to that described, sometimes occurs when it cannot be attributed to mercury. Such an affection in children, under other conditions of the system than fever, has been described by several writers, and appears to have some analogy, in respect to the subjects of it and the course it takes, with the peculiar gangrenous affection of the pudenda which is occasionally witnessed in young girls. We recollect having seen two girls, near Macroom, in the county of Cork, whose faces—that is to say, the lips and cheeks,—were nearly destroyed by, according to the father's account, a gangrenous ulceration which occurred in the course of typhous fever, and which affected also the mother, who died of the disease. These persons had no medical assistance. But, whether or not the mercury had any influence in the production of the gangrene in Dr. Grattan's case, this seems to be certain, that his practice cannot justly be censured; for, as he remarks, we cannot "ascertain *a priori* the existence of such susceptibility with respect to the effects of particular substances;" and, still using his own expressions, "were we to anticipate in every other case a similar result, our practice would become so vacillating and timid, that a great proportion of our patients might die through our hesitation in employing an active remedy until we had first satisfied ourselves, by cautious experiment, that it was not likely to prove injurious."

"*A well-marked Case of Liver-cough, with some Cases and Observations tending to show how frequently the Lungs and other Viscera sympathize with Derangements in the Liver, whether organic or functional,*" by William Brooke, M.D. M.R.I.A. &c. is the title of the next paper.

The case expressly designated above exemplifies, in a very striking manner, the proposition of the author of this paper, besides presenting several other very interesting circumstances. We have, however, extended our account of this volume so far already, that we must confine our extracts to just two or three of the most important points in the first of the cases related by Dr. Brooke. A boy suffered an attack of severe and very obstinate cough, in October 1818, when he was twelve years old, about a month after he had been placed at school. None of the remedies employed—which consisted of bleeding, vesicatories, emetics, purges, sudorifics, expectorants, and opiates,—afforded the smallest relief. After a fortnight, he was recommended to try his native air, and, being taken home, gradually recovered, the cough having continued about five weeks. In January 1819, he returned to school, and was again seized as before, only that the cough was less severe, treated as before, with the exception of bleeding, without apparent benefit; but immediately recovered on being taken home. He returned once more; "but it was only to suffer a third attack of his cough, which, in severity and duration, was nearly equal to the first. A full consultation of the neighbouring practitioners was now called, and every effort made for his relief, but in vain; in eight or ten days he was able to travel, and went home with the advice never to return, as the air was supposed particularly to disagree with his constitution." Early in May he had the measles, with which he coughed much: the cough, however, was such as is usual in that disease, and not at all, as his mother remarked, like the other.

At the end of January in the ensuing year, he was placed at a school in Dublin; and on the 8th of February Dr. Brooke was sent for to see him, from his having been attacked with the cough. This was the first time he was seen, with the cough, by Dr. Brooke. "Upon the most minute and patient examination," he says, "I was unable to detect the smallest deviation from health, or the smallest derangement in any of the organs or their functions, save the cough alone. This was the sum total of all his ailment that I could discover; and certainly it was a most extraordinary cough, and unlike any thing I had ever heard before: he told me it was precisely similar to the former attacks, and appeared quite in despair of being able to obtain any relief. He made a deep inspiration, and then uttered a short cough or ejaculation, sounding somewhat like *haghss*, in an extremely harsh, loud, and disagreeable tone, conveying to the hearer an idea of great distress in the patient, but which really was not the case; as he never complained of any thing until evening, when he always became weary, and felt a general soreness in the muscles of the thorax and abdomen. This monotonous cough was repeated three times very rapidly; he then fully inspired, and coughed as before; and again for the third time inspired and coughed. After this, he became quiet for some time, the interval varying from one or two minutes to four or five; so that each paroxysm may be said to have consisted of three distinct inspirations and nine expirations, the air taken in by each inspiration being expelled by three coughs, or ejaculations, as above described."

He had taken a purgative; and, "to gain time for reflection," Dr. Brooke merely, at first, directed him to remain in bed; to take a mild saline sudorific medicine, as he had been walking on the water-side, during a cold north-east wind, on the day previously to the attack; and to use a tepid pediluvium. On the fourth day, he took three grains of calomel, and two of James's powder, at bed-time; and on the next morning a saline purging draught: after this, he had "several large and very offensive bilious stools yesterday; not the least alteration in the cough; tongue white, and back part thickly furred,—a circumstance which I think I have," Dr. Brooke says, "often observed suddenly to take place in incipient hepatic affections, after a three or four grain dose of calomel; and probably to be accounted for from the peculiar influence of that medicine over the secreting function of the liver, as well as its action on the excretory ducts." On the next day, the patient complained of pain in the right side; and, on careful examination, a tumor, situated below the points of the seventh and eighth ribs, and "occupying a space that might in circumference measure about four inches, was very perceptible to both touch and sight." Leeches and warm fomentations to the side were then employed, and a course of mercury prescribed; by means of which, and a vesicatory, with some other appropriate medicines, he recovered his health. He has returned to school, after having been at home again, and has not experienced any return of the cough.

It should be remarked that this boy, when at home, like most of the youth of the Irish gentry, was "accustomed to a good deal of exercise in the open air, particularly on horse-back," and was then "a hardy healthy boy." He had probably never been at school, or similarly confined, before; for it is very common for the Irish boys to be good hunters before they are able to read.

Had not the subject of cough and more serious affections of the lungs dependant on disease of the liver, been discussed in a general way as it has been by Dr. Wilson Philip, we should, notwithstanding the manner in which we are urged by our limits to close our account of this volume, have adduced some extracts from Dr. Brooke's very judicious and practical remarks on it; but as it is, we can only point them out for the attention of our readers.

An account of *a case of Melæna*, communicated by letter to Dr. Brooke, by Dr. Whitlock Nicholl, in which the spirit of turpentine, a remedy of which the use was proposed and established by the former physician, was successful, occurs next to our notice; and, after this, the history of "*a Case of Ruptured Vagina, which terminated favourably, notwithstanding the Strangulation and subsequent Sloughing of a considerable Portion*

*of the Intestinal Canal;* by THOMAS M'KEEVER, M.D. Assistant to the Dublin Lying-in Hospital."

The patient, 26 years of age, was delivered by the crotchet, after having been in labour twenty-four hours, by a medical practitioner whose name is not mentioned. "This operation occupied fully two hours, and was ultimately accomplished with extreme difficulty, in part owing to the very large size of the child's head, but principally to a deficiency of room in the cavity of the pelvis." On the ensuing morning, one of the patient's attendants observed what proved afterwards to be a portion of intestine, hanging from the vagina; it was then about six inches long: no mention of this was made to the medical attendant. No evacuation from the intestines had taken place by the fourth day, notwithstanding the use of purgative medicines; but there was no nausea or vomiting, nor does it appear that there was tenderness about the abdomen. On the fourth day, one of the women about her pulled strongly at the loop of intestine: after this there came on great pain, hiccough, vomiting, and swelling of the abdomen. Two days afterwards, she was seen, for the first time, by Dr. M'Keever. He found her suffering all that might have been expected under such circumstances, and "near a yard and a half of her bowels coiled up under her, black and, to all appearance, putrid; exhaling a shockingly offensive odour." This portion of intestine separated on the third day from this time, after which the woman had a copious discharge of feces (the first since her delivery) *per vaginam*, and finally recovered her health, with the exception of the evacuation of her feces and urine by the vagina. The portion of intestine is three feet eleven inches in length. It does not appear what division of the intestines it had constituted; but, a month after its separation, it is said that "the stools were of a bright-yellow colour, of fluid consistence, and are altogether free from fecal odour;" and therefore there is reason for conjecturing that it is a portion of the small intestines, as the length of it would also indicate.

"*A Case of Diseased Heart, in a Patient who had suffered severely from acute Rheumatism.* By DANIEL FALLOON, M.D. Licentiate of the King's and Queen's College of Physicians in Ireland."

This case presented the ordinary and severe symptoms of chronic inflammation of the heart, with adhesion of this organ to the pericardium. The interesting and peculiar circumstances which we shall notice, are the great relief from very severe symptoms, indeed from apparent imminent death, and the preservation of the patient's life, in a tolerably comfortable state for the most part, for about eight months afterwards, by means of blood-letting and other remedies directed by Dr. Falloon; but especially, as it appears, by blood-letting, general as well as local, and this very frequently and to a considerable extent. The history of the case is too long for us to attempt to give an abstract of it, but we ought to remark that it presents very favourable indications of the author's talents, and of the energy with which he would employ the means prompted by rational views, under apparently desperate and hopeless circumstances.

The last case in this volume, which is one of *recovery from the effects of corrosive sublimate*, related by CHARLES LENDRICK, M.B. M.R.I.A. is also an interesting one, in consequence of the recovery having taken place after the effects of the poison had been manifested in a severe degree. It appears that the quantity of sublimate swallowed by the patient, who was an adult man, exceeded half a drachm. An emetic had been administered, and vomiting had taken place, before he was visited by Dr. Lendrick, who saw him with Mr. Buchanan. Orfila's remedy, the whites of eggs beat up with water, was then administered; and it was found, on visiting him a few hours afterwards, that "the alarming symptoms had diminished after the first exhibition of the eggs, and almost entirely subsided subsequently to the others."

From this time his recovery was progressive; but it is interesting to remark, "symptoms resembling periostitis occurred over each tibia about the tenth day, and it was proposed to make an incision: as, however, he was now sufficiently recovered to attend to his ordinary avocations, this intention was relinquished, and the use of the warm-bath proved sufficiently effectual. A slight degree of paralysis of the right side, with nervous irritability in other respects, attended with some loss of memory, supervened; and from these complaints he is not yet free." The paper is dated at the period of four months from the occurrence of the accident.

---

*Practical Observations in Midwifery; with a Selection of Cases.* Part I. By JOHN RAMSBOTHAM, M.D. Lecturer on Midwifery at the London Hospital, and one of the Physician-Accoucheurs to the Lying-in Charity for delivering poor Married Women at their own Habitations. 8vo. pp. 422. T. and G. Underwood, London. 1821.

MIDWIFERY is a department of the art of medicine in which books of the character of that before us are of especial utility, —it may, indeed, be said, of absolute necessity. General principles or precepts may direct us, with tolerable precision and success, in the treatment of most diseases; but, in the more complicated and difficult cases of parturition, the practitioner seeks, with anxiety, for precedents which furnish examples of what nature has accomplished, and of what has been effected by art, on, as nearly as possible, similar occasions: he feels the insufficiency of general principles, which may not take especial cognizance of minute circumstances, that, in certain cases, become matters of paramount importance, though ordinarily of but trivial interest; and he knows that, in cases of imminent danger, there is not time for that reflection which might enable him to apply those principles to the occasion before him with due confidence and precision, even though they embraced all the indications which should regulate his conduct. Those remarks might, it is true, be almost equally well applied to some points in surgery; and hence it is that we all of us perused, in the course of our studies, the writings of POTT on Injuries of the Head with so high a degree of interest and gratification. This allusion to those Dissertations of Pott has occurred somewhat luckily for us, as it points out the means by which we may, in a few words, enable the reader to form a notion of the character of the work before us, by stating that the plan and method of the two are similar, only that Dr. Ramsbotham has preluded his cases illustrative of certain points of physiology or practice with general considerations adapted to a complete series of cases, in an uninterrupted manner; whilst Pott has often treated of the several points of his cases in distinct and separate discussions. The addition of these preliminary general reflections gives the work of Dr. Ramsbotham a decided superiority, as regards its method alone, without considering the value of the accounts of cases, to that of Smellie and other collections of observations of a similar kind; as it combines the utility of general principles with that of particular examples.

The subjects treated on in this volume are, in the first place, the physiology of the uterus as far as regards the parturient function; the phenomena of natural labour, and the conduct of the medical assistant, especially in respect to the management of the placenta; and the ordinary "occurrences after delivery:" after which are considered, in succession, *adhesion of the placenta*,—*retention of the placenta*,—*disruption of the placenta*,—*relaxation of the uterus after delivery*,—*collapse after labour*,—*protracted labours, with their different causes and degrees of difficulty*,—and *rupture of the uterus.* The author says, in the Preface, "Should the present attempt be favourably received, I may be induced, at some future time, to continue and extend practical observations to other cases of difficulty and danger in the act of child-birth."—"I have merely stated such facts as I have seen, (he also observes;) such, indeed, as have occurred in my own practice: I may have omitted many remarks which ought to have been inserted, but I have not availed myself of the writings of others." This, we believe,—considering the writings on Midwifery that were already extant,—is the best way in which the author could have presented to the public the results of his observations and reflections. It is particularly interesting to practitioners to know what are the most important cases of difficulty which have occurred to one man in the course of his life, and what inferences such cases have led him to form; as those which have happened to one—bating only what depends on the age and particular opportunities for observation of the individual in question—is really likely to happen to every one; and the inductions made by a man of good talents from what he has himself observed, respecting such a subject as that under consideration, are of very different and superior value to those founded, to more or less extent, on the recorded observations of others.

It does not come within the province of the Reviewer to give a regular analysis or abstract of a work like this; we have only to remark, in a general allusion, that the author's reflections are characterized by good judgment and comprehensive views, and present, in his several discussions (already enumerated), excellent practical dissertations on the subjects to which they relate; whilst the cases which are narrated are, for the most part, very interesting, and strikingly illustrative of the points to which they are applied. We shall not have any of them transferred to our pages, with the view—as it is said by the followers of this trite practice—*of giving a specimen of the author's manner;* for, in truth, to divulge "a secret of our prison-house," this is only the Reviewer's excuse for filling a few pages with no other trouble to himself than the sending a direction that such a passage should be copied, with the book, to his printer. There are many books, of which it is possible to give a tolerably satisfactory view in a concise abstract, but this is not the case with one of the character of that before us, where the utility of it consists chiefly in the minute and precise details which it comprises; and, if the work possess the merit and utility we believe it to possess, the selection of a detached part would be as absurd an attempt to show it, as that of the Grecian fool who carried with him a brick as a specimen of the house he had for sale. Of the author's observations on *rupture of the uterus,* we shall, however, give an abstract, and connect it with some observations contained in a dissertation by Dr. DEWEES;* which, with the evidence to which we shall also refer, seem to us to be

* Published in the second Number of the *Philadelphia Journal of the Medical and Physical Sciences.*

fair and forcible arguments against the precept of HUNTER, GARTHSHORE, and DENMAN, for the treatment of cases in which such an accident has taken place, and which has hitherto favoured the practice, generally, in England, that resulted also from the example of SMELLIE; who says, in reference to a case of rupture of the uterus which he had met with, "In order to avoid reflections, this accident was kept secret."*

Dr. Ramsbotham relates here seven cases of rupture of the uterus,† which he has himself witnessed, and which occurred either at the full period, or near to it, of ordinary utero-gestation. The volume contains, besides, an account of a case where this accident occurred about the fourth month; and another, which has been communicated to the author. Every case which has occurred to the observation of Dr. Ramsbotham "has sooner or later proved fatal."—"Some women (he remarks) scarcely survive delivery; others bear up against the effects of the accident for several days." Some writers, as CRANTZ and LEVRET, have pretended that the occurrence of this accident might sometimes be foreseen, by certain signs; but these are extremely equivocal, in the opinion of Dr. Dewees, who has minutely examined them; and Dr. Ramsbotham says, "I know of no particular symptom threatening its approach, or indicating when it is about to happen, which would justly warrant a premature resort to delivery." We shall transcribe Dr. Ramsbotham's account of the signs of the actual occurrence and effects of this accident.

"Rupture of the uterus always takes place suddenly, and generally without any previous warning. While the labour appears to be going on naturally but slowly, the woman is seized in the middle of a strong expulsive effort, with an uncommon pain in some part of the

---

* Smellie, it is worthy of remark, seems to have felt no repugnance on finding his example followed by others; for he publishes an account received from a correspondent, who tells him, in a similar case, that, "according to his *prudent* advice, he spoke nothing of the matter." A disposition to follow this *prudent* advice has conduced, perhaps, to the opinion, which has not unfrequently been expressed, that rupture of the uterus is but a rare accident in parturition, since so few instances of it have been recorded, at least in English medical literature. But, when we consider that Dr. Ramsbotham has been called to nine cases, and that GREGOIRE of Paris witnessed sixteen in the course of thirty years' practice, it would appear to occur not very unfrequently. Though, on the other hand, it is stated that, amongst twenty-three thousand one hundred and fifty child-births, which occurred in a given time at the Hospice la Maternité at Paris, there was only one case of rupture of the uterus. It is not at all probable that the accident could have happened in that establishment, at least if death ensued from it, without the discovery of it. This evidence is worthy of being collated with the fact that the greater proportion, by far, of the recorded cases which have occurred to the observation of medical men, have affected women who have been attended in their labour by midwives alone, or by obviously unskilled practitioners, until the occurrence of the accident.

† Two other cases are also related by Dr. R. in the London Medical and Physical Journal, for October 1813 and September 1814.

belly: this pain is of a very different nature from those pains of labour under which she has hitherto suffered; she has never felt the like in any preceding confinement. The attack of this new pain usually occasions a shriek, and is accompanied with the sensation of something having given way within: it is commonly followed by a sense of weight and oppression, and sometimes by the feel of a rising of her *burthen*. The patient now involuntarily puts her hand to her belly, with a complaint of increased suffering, and utters frequent exclamations expressive of misery, with "Oh! this pain!" This new pain is referred to one point, on one or other side of the uterine tumor; and it is stated to be similar to that which would be occasioned by cutting or tearing the parts asunder, and sometimes it is likened to the cramp. After its attack, the regularity of the labour-pains is suspended: uterine action either ceases altogether, or is gradually diminished in energy and effect. By and by, the woman complains of faintness, which shortly approaches to syncope; the countenance becomes pallid, and is at the same time expressive of great anxiety; the eye rapidly loses its natural lustre; the pulse gradually gives way, and becomes quick and tremulous; difficulty in respiration is presently perceptible in a greater or less degree; and there is a general restlessness of body, with coldness of the extremities. In cases in which there has been no previous sanguineous discharge, a slight degree of external hemorrhage now makes its appearance. In those in which there has previously been some trifling show, it is suddenly increased in quantity. Vomiting of greenish or dark-coloured fluids, in some instances, almost immediately supervenes to the accident; in others, it comes on a short time before the death of the patient. There is an occasional return of uterine action, but in a slighter degree, which the woman unavailingly assists by the voluntary efforts of the diaphragm and abdominal muscles: she is at the same time perfectly aware that there is a material alteration in the kind of pain, from her inability to bear down as she has been accustomed to do."

"A rupture of the peritoneal coat of the uterus," Dr. R. adds, "sometimes happens without extending itself into the uterine structure. Under this occurrence, we observe all the symptoms of actual rupture of the uterine structure itself, in a diminished degree, except those connected with the escape of the child.

"A breach in the vaginal surface also occasionally occurs, which seems to be produced by the continued pressure of the head, impelled by powerful uterine action. If the breach be trifling, the accident may not be productive of much inconvenience: if it be extensive, and especially if an opening be made into the abdominal cavity, such a similarity of symptoms follows as induces a suspicion that the uterus has given way."

A very remarkable case of rupture of the peritoneal tunic alone, is related by Mr. C. M. Clarke, in the third volume of the Medico-Chirurgical Transactions. This case terminated fatally half an hour after the accident, (the woman dying undelivered, under the care of a midwife,) having presented the ordinary symptoms of rupture of the uterus generally.

Dr. Ramsbotham says he has never met with rupture of the uterus in a first parturition.

"The accident has happened, in those cases which I have seen," he says, "in a subsequent labour, and sometimes after several difficult births, though living children have been expelled. I am thence led to suspect, either that the uterus has received some local mechanical injury from the violence of its own efforts, or from the previous effects of artificial assistance, by which its structure is at this point weakened; or that it is thinned at the part where it gives way, during the last months of gestation, by continued pressure against some prominent part of the pelvis.

"The breach of structure usually happens somewhere about the cervix, either anteriorly towards the symphisis pubis, or posteriorly towards the prominence of the sacrum. The rent is either transverse or is carried laterally upward. The fundus uteri rarely gives way, yet its body and sides occasionally do."

Dr. Dewees has arranged all the obvious causes of this accident under two heads: those which act directly, and those which act indirectly, on the uterus.

"The first, or direct, are mechanical violences, and may be external or internal. The external may be a blow, a fall, a kick, or violent pressure; the internal may be, attempts to turn, or to return a prolapsed limb, or the mal-adroit application of instruments, or the unequal surface the fetus itself may present.

"The second, or indirect, are those which impair the integrity of the substance of the uterus; such as all those causes which offer a mechanical impediment to the passage of the child, as a contracted pelvis, an unusual sharpness of the linea iliopectinea, and exostoses, tumors, scirrhous indurations, and ulcers."

In a case which occurred to the observation of the physician just mentioned, the influence of inordinate pressure during the course of utero-gestation, seems to be well proved: the head of the fetus measured one foot ten inches in the horizontal circumference of the superior part of the cranium, "and the lower part of the wound, (in the uterus,) where the rent began, was gangrenous." This gangrene cannot be supposed to have commenced subsequently to the rupture of the uterus, for it does not appear that the woman lived more than twelve hours after that accident; but, as it was not accompanied with any striking symptom, it was not known precisely when it happened, though it seems probable that it occurred only a few hours, at the utmost, previously to death.

Dr. Ramsbotham is disposed to think that a "*thinning*" of some part of the uterus, from undue pressure during pregnancy, may be a common cause; and Mrs. Boivin mentions that, in a woman who died soon after delivery at the Maternité, from a pulmonary affection, whose pelvis measured only two inches and three quarters from pubis to sacrum; the uterus, at a few lines above its neck, in the situation corresponding with the sacro-vertebral angle, appeared to be very nearly *worn through* for the space of two-thirds of an inch, having in this part not above the eighth part of a line (96th part of an inch) in thickness. The fetus had been extracted by means of the perforator. The cases of DENMAN, BYE, and several others, show also the effect of undue pressure on some projecting point of the bones of the pelvis, in the production of this accident.

One of the cases related by Dr. Ramsbotham, in which the rupture took place about the fourth month of pregnancy, is particularly remarkable. Dr. R. says,

"About four *p.m.* on Friday, June 2d, 1820, I was called in a hurry to see a lady in Providence-row, Finsbury, who was stated to be dangerously ill, and who was said to be about four months advanced in pregnancy of her first child. I learnt that she had been suddenly seized with sickness and vomiting about eleven *a.m.* (after passing a good night,) which her friends attributed to some mackerel dressed with vinegar, of which she had freely eaten the preceding evening at supper. The family apothecary had been called, who ordered some medicine; but, as she seemed to be getting worse hourly, I was sent for. I found her under symptoms of the greatest danger: her pulse was scarcely to be felt; her countenance was pallid and depressed; her hands were clammy and cold; and she complained of pain in the belly. There had been no external flooding, yet the symptoms struck me as being indicative of internal loss of blood or of the effects of lead on the constitution. I ordered some opening medicine, and the frequent injection of clysters, with a promise that I would shortly see her again. In little more than an hour a message was sent to my house, requesting I would see her again immediately: on my arrival at the house, she was dead. Leave was obtained to inspect the body the next day. The uterus was found to be ruptured on its left side, and the ovum had escaped in its membranes entire into the cavity of the abdomen, in which was also a large quantity of coagulated blood, to the amount of several pounds. The uterus had a singular appearance: it seemed double, and to consist of two parts united longitudinally together; but the ruptured portion had no external opening,—that is, it had no os uteri. Each portion had an ovarium attached to it. This uterus and ovum are preserved."

A case nearly similar to the foregoing is related by DIONIS: the remarkable difference between the two consisting in the communication of the cavity of the uterus in which the fetus was lodged with the vagina in this, whilst it was wanting in that of Dr. Ramsbotham. The subject of the case mentioned by Dionis was a *femme de chambre* of a dauphine of France: she was seized, towards the sixth month of her pregnancy, with violent pains in the belly, which continued for three or four hours; after this she, for a time, ceased to feel the motions of the fetus. Twelve days afterwards, she was taken, about eight o'clock in the evening, with pains not less severe than those in the former attack; she now and then experienced nausea and vomiting. Convulsions came on in the course of the night, with cold sweats, tumefaction of the belly, and great prostration of strength, which terminated in death. Dionis opened the body. He found the fetus amongst the intestines, immersed in a large quantity of blood. The navel-string was whole, and the placenta still adhered to the uterus. This organ was divided, towards its fundus, into two parts, each having a distinct cavity, both of which had a common opening into the vagina. Each body had a fallopian tube and ovary: the left one, which had contained the fetus, was ruptured; the right presented the produce of a more recent conception, which was of the size of a small egg.

The most extraordinary accident of this sort on record, is that related in one of the volumes of the Edinburgh Medical Essays, where not only the uterus, but the abdominal parietes also, burst, and exposed the fetus.

Although all the cases which have occurred to the observation of Dr. Ramsbotham have terminated fatally, he says, "Some cases are upon record, in which the woman has recovered. Notwithstanding my want of success, I have always thought it my duty to offer a chance of life to the mother, by the only practical expedient,—by as early a delivery after the accident as the case would allow. I cannot accede to the doctrine of allowing the woman to die undelivered."

The establishment of this rule of practice is the especial object of Dr. Dewees, in the dissertation to which we have already referred; and, after a minute, candid, and judicious examination of the histories of cases on record, he seems to have shown its propriety in the most satisfactory manner. The arguments used by Hunter and Denman, for the contrary practice, are discussed by him in an extensive manner, and he finally "challenges the advocates of Dr. Denman's opinions to prove that there was an instance of recovery, 'where no operation was performed;'" whilst many well-authenticated instances of recovery when delivery has been effected, are recorded.

"The first instance that is distinctly recorded," says Dr. Dewees, "is that mentioned by Heister,* on the authority of a surgeon named Rungius. In this case, the intestines were distinctly felt through the rupture of the uterus, and through which the fetus was extracted; yet

* *Instit. de Chir.* tom. ii. p. 137.

the woman recovered. Dr. Douglass, in his essay,* gives the history of Mrs. Manning, who also recovered. Dr. Hamilton† relates another instance of complete and entire recovery, although the intestines issued through the wound of the uterus, and were reduced by him after the delivery of the child. In this case, he declares "the recovery was nearly as good as if no extraordinary accident had happened." Dr. Ross relates the case of a Mrs. Granan, of Eppendorf, near Hamburgh, who suffered this accident in two consecutive labours, and yet recovered.‡ Mr. Kite gives a case of ruptured uterus, which terminated favourably.§

"In a copy of the MS. Lectures of Dr. J. Hamilton, the present Professor of Midwifery in Edinburgh, there are two cases related of recovery; one of which he himself attended, and says, it was 'one in which almost every circumstance was unfavourable;' for, 'in bringing the child through the lacerated part,' he 'felt the uterus tearing more: the woman lost three pounds of blood; yet she recovered, and afterwards had children." The other case occurred in Lancashire:— 'A poor woman fell from a cart, in consequence of which the uterus was ruptured, and the child passed into the abdomen. The bones of the pelvis were so much injured by the fall, as not to allow of delivery,

* "*Essay on the Rupture of the Uterus*, p. 7.

† "*Outlines*, p. 344.

‡ "*Annals of Medicine*, vol. iii. p. 377.

§ "*Mem. Med. Soc. of London*, vol. iv. p. 253. Madame La Chapple also; *Annuaire Medico-Chirurg.* tom. i. p. 542."—We have to remark respecting the case related by Mrs. La Chapelle, that it is not so precisely applicable to the question under consideration as Dr. Dewees intimates, by the context. It was not a well-determined case of *rupture of the uterus*, by which the fetus might have escaped into the abdominal cavity: it seems to have been rather a separation, to a certain extent, of the uterus from the vagina, by a transverse laceration, or such an accident as is noticed by Dr. Clarke, of Dublin, in the following paragraph, referring to the cases detailed in the Transactions of the Association of Physicians of Dublin.—"A survey of these eight cases, (Dr. Clarke says,) will show that the anterior part of the vagina, near to its connexion with the os tincæ, is the part most apt to give way on certain extraordinary efforts, whether of nature or art:" only that the separation took place at the *posterior* part of the vagina, in the case of Mrs. La Chapelle. We quote her account of it. "On the introduction of a finger into the vagina, I perceived that this canal was separated from the neck of the uterus at its posterior part. The state of the patient did not permit a more full examination; we only directed our efforts to the recovery of her strength."

Dr. Dewees says, (we may remark in this place,) in another part of his dissertation, "If the laceration happen to the neck of the uterus, or at its connexion with the vagina, it is much more frequent that the fetus, with the placenta, pass immediately into the abdomen." This inference is precisely opposite to that which we should be disposed to form, from reasoning on the mechanism of the parts concerned; and it is exactly opposite to the conclusions of Portal, who says, "Il paroit, d'aprés la lecture de diverses observations rapportées par les auteurs, sur la rupture de l'uterus pendant l'accouchement, que lorsqu'elles se sont faites dans son fond, ou dans son corps, l'enfant s'est frayé une route dans la cavité du bas-ventre, et que la femme à peri; mais que, lorsque c'est le col de la matrice qui s'est dechiré seulement, alors l'accouchement s'est fait par les voies naturelles, et la mère a pu vivre." Saviard and Chaussier make similar remarks. Rupture at the connexion of the uterus and vagina is, according to most authors, very frequently owing to improper or awkward introduction of the hand.

being much *mashed:* the Cæsarian operation was performed, and she recovered.'

"Mr. Thibault* relates a similar case to the one just recited; gastrotomy was performed with the most entire success to the woman, though too late for the preservation of the child.

"Baudelocque relates† that a M. Lambron, a surgeon of Orleans, performed gastrotomy twice on the same person, with the desired success to the woman, after the rupture of the uterus. This woman became pregnant a third time, and was delivered naturally of a healthy child.

"Mr. Hugo‡ relates a successful case also, and we could without difficulty increase the number; but these are sufficient to prove that success has attended the 'interposition of art.' The cases we have just cited were all fortunate to the woman, but the child uniformly perished: this was rather owing to the time at which art interfered, than to the mode it adopted. Of this we have sufficient proof in the case related by Burton.§ He says, 'I was called to the wife of a broker in the city of York, who had had several children: she fell into labour at the regular time; she had only a slow labour at first, but after some little respite her pains became more violent; during one of which she perceived something to crack within her, as she termed it; after which, she exchanged her pains for faintings, &c. with an intermitting pulse. On this account I was called in. Being told every thing that had happened, I was apprehensive of what indeed proved to be the case: wherefore I told the by-standers my opinion; and that, as the child was alive, it was proper the woman should be delivered as soon as possible; which was done directly. The child was small, but very healthful and lively. Immediately after the birth, I introduced my hand into the uterus, where I found one side of it burst so wide as to have admitted my hand to pass through the opening.' Mr. Haden relates a case that terminated with safety to both mother and child.||"

In addition to those, we are enabled to add the cases related by Dr. Clarke, of Dublin;¶ Dr. Labatt;** Dr. Frizell;†† in the Gazette de Medecine for 1778, (where the child was turned and delivered by the feet;) and by Dr. Rossi, of Parma, noticed in a late volume of this Journal. In the last instance gastrotomy was performed. The Leipzig Commentaries also contain the history of a case, where the uterus was ruptured by a violent blow on the belly: the fetus was felt projecting at the

* "*Jour. de Med.* for 1768. † Heath's *Translations*, vol. iii. p. 430.
‡ "*Med. and Phys. Jour.* for March 1808.
§ "*System of Mid.* sec. xliii. p. 110.
|| "*Med. and Chirurg. Trans.* vol. ii. p. 118."
¶ *Trans. Assoc. Dub. Coll. Phys.* vol. i. We should however remark, that this case is not satisfactorily shown to have been an instance of rupture of the uterus, or that it was more than one of rupture of the vagina, or of the connexion between the vagina and uterus.
** *Dublin Medical and Physical Essays*, vol. i. †† *Trans. Assoc. Colleg.* vol. ii.

left side of the abdomen; an incision was made here, and the fetus extracted. The woman afterwards bore children, in the ordinary way.

Dr. Dewees endeavours to show that the instances of supposed recovery from the effects of this accident, when the case has been "resigned to the natural efforts of the constitution," are not valid examples of such an event: for that it does not appear certain that the uterus was completely ruptured; or, in other words, he thinks some of them have been cases of original extra-uterine gestation, and others of rupture of the rest of the uterus whilst its peritoneal covering has remained entire, or that the fetus has not really passed into the cavity of the abdomen, but been enclosed in a membranous cyst formed by the peritoneum. We think it probable that Dr. Dewees is correct in supposing that some cases have been instances of extra-uterine gestation, in which the attendants have been deceived by the occurrence of the ordinary pains, like labour-pains, about the usual period of utero-gestation, which are known to occur in such cases, and continue for several hours, as we had occasion to remark in some late Numbers of this Journal.* Dr. Dewees notices particularly the cases said to have been instances of recovery when the fetus has escaped from the uterus into the cavity of the abdomen, and suffered to remain there, that have been related in the Journal de Medecine for 1780, and that by Dr. Sims.† That in the Journal de Medecine is selected, he says, as "one of the most favourable cases we could find upon record for the above opinion." We cannot transcribe his discussions of these points; they are necessarily too long for the limits of our Journal. In support of the opinion that the peritoneal coat of the uterus may have formed a cyst for the fetus in the cases alluded to, Dr. Dewees says, "We have Dr. Ross's third case, (Annals of Medicine, vol. iii. p. 306. 'On opening the abdomen of this cadavre,' says Dr. Ross, 'it was found under the ligamentum latum of that side, (the left,) an arm of the child could be felt, covered only by the peritoneum.' Here then is demonstration that the substance of the uterus can be torn without doing violence to its peritoneal covering."

Dr. Dewees will, probably, be thought to have formed too exclusive an opinion respecting the instances of recovery from the immediate effects of the accident, when the case has been left to the natural efforts of the constitution, and the fragments of the fetus have, at some remote subsequent period, been evacuated through the abdominal parietes. It must, however, be

---

* No. 248, p. 345; and vol. xli. p. 514.

† *Medical Facts, &c.* vol. viii.

very difficult to prove that, in any of these or similar cases, the fetus has been really in the abdominal cavity,—that is to say, not enveloped in a particular cyst; because the only evidence that is valid must be obtained by examination after death; and, on the patient surviving for any considerable period, there will, in all probability, in every instance, (as we observe in all analogous cases,) be something like a cyst formed around it, by means of the coagulable lymph effused during the inflammation which must ensue from the presence of such a foreign body in the cavity of the belly; or else so much alteration of the parts concerned will have taken place, that it will not be possible to decide whether or not a cyst had ever existed. Dr. Dewees, however, endeavours to prove that the cyst which was present in the case related by Dr. Sims, was not formed in this way, but was the natural peritoneum. However, admitting these cases to be instances of what is above denied, it does not appear that near so many of them can be cited, as there can of those of recovery when delivery has been effected by art. We have, besides, to take into consideration the wretched condition of those women who have survived the immediate effects of the rupture, and carried about with them a fetus undergoing decomposition in the abdomen. Many, indeed most, of those, on the other hand, who have been delivered, have subsequently enjoyed good health, and some of them have afterwards borne living children; as will be seen by referring to the cases noticed by Dr. Dewees, in a passage which we have already transcribed.

The remarks of Dr. Denman on this subject are absolutely surprising; he says, there was no instance of recovery within his knowledge, "except one, *which was doubtful*, of either of them (mother or child) being preserved,"* when delivery had been effected by art. This remark is made several years subsequently to the time when he had said,† "Besides some few others, of which I have been informed, or which are recorded, a case has occurred to my very worthy, able, and experienced friend, Dr. Andrew Douglass, in which the uterus was ruptured: he turned the child, the patient recovered, and had afterwards children. If no other case," he continues, "had ever occurred, I apprehend this would be sufficient authority to render it in future the indispensable duty of every practitioner to act in a similar manner; and, bad as the chance is of the patient, to be strenuous in using all the means which art dictates to extricate her, if possible, from her danger, or to preserve the child."

* *Essay on the Rupture of the Uterus.*

† In his *Introduction to Midwifery*, vol. ii. p. 117.

Dr. Denman may be readily excused for altering his opinion respecting the practice to be pursued in such cases, between the time of the publication of the two works referred to, and for being ignorant of some recorded cases of recovery, (if not in England, in other countries,) or for judging differently from most other men respecting their validity; but his attempt to throw suspicion on the case of his "*worthy, able, and experienced friend, Dr. Andrew Douglass,*" is not too harshly designated by Dr. Dewees, when he terms it "*disingenuous.*" Dr. Dewees also, very properly, says, "Why the case of Dr. Douglass should be doubtful in the year 1810, when it was recorded as an unequivocal instance of recovery in 1795, is extremely difficult to tell: it looks too much like a subterfuge to avoid the force which the fact brings with it."

Besides the preponderance, at least, of the instances of recovery when delivery has been effected by art, Dr. Dewees shows that the women who have been thus delivered have, on an average, lived longer after the accident than those who have died undelivered; and, probably, most practitioners may have been disposed to say, after thus using their best efforts, though unsuccessful, for their patients, with LAMOTTE, "Quelqu' inutile que fût cet accouchement, nous fumes plus contens tous deux, elle d'ètre accouchée, parce qu'elle en mourut plus tranquillement, et moi de l'avoir executé."*

In a case of this accident, though delivery by art be determined on, the question, in what way delivery is to be effected, is still to be settled. The obvious means are, turning and delivery by the feet, delivery by the crotchet, and gastrotomy. The choice has but very rarely fell on the last method: but, in respect to this point, Dr. Ramsbotham remarks—

"As the number of women who have ultimately recovered from this accident is at the present so trifling, and as the occurrence is in itself almost necessarily fatal to the mother, it may be a question worthy the consideration of the profession, whether the Cæsarian section, offering a mode of freeing the mother from the child, with a chance of its life, ought not occasionally to be substituted for the perforation of the head. But, in determining on this tremendous expedient, which will place the chance of recovery to the mother in a still lower scale, we ought previously to ascertain, if not to a certainty, as far at least as probability will allow, that the child is still alive under the breach in the uterine structure. If this be the case, such a length of time ought not to be allowed to pass away in the interval as can be supposed to interfere with that life."

* The French practitioners, subsequently to the time of Lamotte, as well as those of the continent generally, it may be right to remark, make no doubt of the propriety of using the most prompt and efficacious measures for the delivery of the fetus.

We shall also transcribe the remarks of Dr. Dewees on this point, as he supports his arguments by precise references to facts; and to the successful cases of this operation which he notices, we may add that related by BAUDELOQUE (*Rech. sur l'op. Cæsar.* p. 58,) and that noticed in a late volume (xli. p. 514,) of this Journal, by Dr. ROSSI.

"Gastrotomy and the Cæsarian section present horrors to the mind peculiarly their own; nor should we be able to overcome the appalling sensations they produce, if we were not influenced by paramount considerations. To save life is a strong motive to the operation; and to be snatched from death is a powerful inducement to submit to it. Where this is the only resource, the case should be fairly and candidly stated, that no after blame may attach; and, in all cases of such hazard, responsibility should be divided, by requesting the concurrence of a brother practitioner, where time too precious would not be lost in this compliance. It has been called a 'horrible expedient' by Dr. Douglass.* It is so confessedly; so are lithotomy and many other operations: but this is not to be the test. Its utility alone ought to determine whether it should be considered as a resource of our art, or be for ever proscribed: for we are by no means satisfied with Dr. Douglass's reasoning upon this subject. He asks, 'If a rupture of the uterus is of itself an injury so generally fatal, what is the patient likely to gain by combining the dangers of such an accident with those of a penetrating wound which will expose the abdominal viscera?'† Dr. D. appears to have forgotten that there was already 'a penetrating wound' which 'exposed' the abdominal viscera; and that an additional one through the integuments would scarcely enhance the risk, since we know that wounds of this kind are not necessarily mortal.‡

"Besides, what would Dr. D. have us do in those cases where there is no possible alternative (as where the pelvis is much deformed,) but this operation? for it is only in cases similar to these that the operation is recommended. The woman can but die after the operation; and this she certainly will do if it be not had recourse to: and what practitioner would not prefer an alternative that may succeed, though hazardous, to the abandonment of a patient to the unrestrained consequences of disease?

"That it has been successfully employed, we are not at liberty to doubt; nor is there any testimony that it has been either wantonly em-

* *Essay*, p. 51. † Ib.

‡ "We find three highly interesting cases of the extirpation of the ovaria, in which there was a speedy restoration to health, although the wounds through the teguments of the abdomen were extensive, and its cavity a long time exposed to the air. In neither of these cases did any untoward symptom arise; though in the first case the tumor was so large as to contain fifteen pounds 'of a dirty glutinous-looking substance,' and the sac which contained it, after being extirpated, 'weighed seven pounds and one half. In the second, notwithstanding every care was taken to prevent it, a quart of blood was spread among the intestines; yet no unpleasant symptoms are said to have arisen. In the third, a diseased ovarium was taken out, which weighed six pounds; yet the patient recovered 'in two weeks.'—Dr. M'DOWELL's Cases, *Eclectic Rep.* vol. vii. p. 242.

ployed, or that it has added new sufferings or new dangers to the already almost-certainly fatal disease for which it is proposed as a remedy. We shall add the evidence we are in possession of, that it has been successfully performed, and from it allow every one to draw his own conclusions as to its advantages. As regards ourselves, we have no hesitation in believing that it is exclusively indicated in several combinations of ruptured uterus. Mons. Thibaut des Bois, a surgeon of Mans, has given an account of this operation having been successfully performed on a woman several hours after the accident, though too late to benefit the child. He adds, 'that the woman suffered scarcely more than from the consequences of a common labour.'* M. Lassus† quotes a history of this operation having been twice performed with entire success on the same woman."

Dr. Ramsbotham says, " In all cases there is a narrowness, if not an absolute deformity of the pelvis, so that perforation of the head becomes, too commonly, indispensably necessary to the delivery." This remark is certainly not universally applicable : it is probable it is not to the cases where women have borne living children subsequently, (at least, and perhaps to several of the others ;) and it is certain that it is not to the case which occurred to the observation of Dr. Dewees, already noticed, where there was " neither 'deformity of pelvis,' 'exostosis,' nor unusual 'sharpness of the linia iliopectonea ;' no 'tumor,' 'schirrous induration,' nor 'cartilaginous condition of the os uteri.' " In continuation from the passage last transcribed from the remarks of Dr. Ramsbotham, we find him say, " If the presenting part of the child have retreated from the situation which it had previously occupied, so that a considerable portion of the child has escaped into the abdominal cavity, delivery must be effected by the introduction of the hand, and extraction by the feet."

---

* *Journal de Med.* for 1768.

† *Pathologie Chirurgicale*, par M. Lassus, tom. ii. p. 237.

